# Analysis of Volatile Organic Compounds Emitted by Plant Growth-Promoting Fungus *Phoma* sp. GS8-3 for Growth Promotion Effects on Tobacco

**DOI:** 10.1264/jsme2.ME12085

**Published:** 2012-10-19

**Authors:** Hushna Ara Naznin, Minako Kimura, Mitsuo Miyazawa, Mitsuro Hyakumachi

**Affiliations:** 1 The United Graduate School of Agricultural Sciences, Gifu University, 1–1 Yanagido, Gifu City 501–1193, Japan; 2 Laboratory of Plant Pathology, Faculty of Applied Biological Sciences, Gifu University, 1–1 Yanagido, Gifu City 501–1193, Japan; 3 Department of Applied Chemistry, Faculty of Science and Engineering, Kinki University, 3–4–1 Kowakae, Higashiosaka-shi, Osaka 577–8502, Japan

**Keywords:** *in vitro*, *Phoma* sp. GS8-3, plant growth-promoting fungus, tobacco, volatile organic compounds

## Abstract

We extracted volatile organic compounds (VOCs) emitted by a plant growth-promoting fungus (PGPF) *Phoma* sp. GS8-3 by gas chromatography and identified them by mass spectrometry. All of the identified compounds belonged to C4-C8 hydrocarbons. Volatiles varied in number and quantity by the culture period of the fungus (in days). 2-Methyl-propanol and 3-methyl-butanol formed the main components of the volatile blends for all the culture periods of fungus. Growth-promoting effects of the identified synthetic compounds were analyzed individually and in blends using tobacco plants. We found that the mixture of volatiles extracted from 3-day-old culture showed significant growth promotion in tobacco *in vitro*. The volatile blend showed better growth promotion at lower than higher concentrations. Our results confirm the potential role of volatile organic compounds in the mechanism of growth enhancement by GS8-3.

Plant growth is influenced by an abundance of abiotic and biotic factors. Plant growth hormones dominatingly affect plant growth, whereas the photosynthetic rate is dominated by temperature, irradiance and gaseous atmosphere (16). These physiological functions have been utilized as classical plant growth regulators; however, along with the composition of the nutrient medium, the composition of the gaseous atmosphere is another important factor for proper growth and development of plants (3). Several gaseous components are present in the atmosphere, especially nitrogen, oxygen, carbon dioxide and different types of volatile compounds produced by surrounding organisms, including the plant itself (5, 37). Changes in these components during different physiological functions *in vitro* largely affect the photosynthesis and other biological functions of the plant (5).

Recently, it has been demonstrated that plants have evolved the capacity to release and detect volatile organic compounds (VOCs) in their environment, and plant growth is promoted by VOCs from beneficial microorganisms (32, 44). VOCs, the major source of secondary metabolites and important components in ecosystems (2), are intensively studied due to their availability as a biocontrol resource. VOCs characterized by low molecular weight and high vapor pressure are produced by all organisms as part of their normal metabolism, and play important roles in communication within and between organisms (34). VOC-mediated interactions among plant–plant, plant–insect and bacteria–plant have been frequently documented (8, 10, 19, 32, 35). Plants also perceive the presence of pathogenic microbes via metabolites derived from the pathogen and activate defensive responses against the pathogens (1). Although the details of the molecular interactions are currently unknown, low-molecular-weight plant volatiles such as terpenes, jasmonates and green leaf components have been identified as potential signal molecules for the plant (11, 13). Koitabashi (22, 23) reported that a filamentous fungus isolated from the wheat leaf produces volatile materials that could suppress diseases and promote the growth of different plants. Subsequently, volatile-producing fungus *Muscodor albus* was reported to have the capacity of growth enhancement and biological control of soil-borne diseases (29). Although the signaling network between plants and microbes has been extensively studied for the past 20 years, little is known about the role of microbial VOCs in regulating plant growth and development.

Currently, beneficial micro-organisms are increasingly used as inoculants for biofertilization, phytostimulation and biocontrol, because reduced use of fertilizers and fungicides in agricultural production is necessary to help maintain the ecosystems and to develop sustainable agriculture. The use of both bio-fertilizers and biocontrol systems can have minimal effects on the environment and such strategies have been widely researched. Plant growth-promoting rhizobacteria (PGPR) and plant growth-promoting fungi (PGPF) are naturally occurring soil microorganisms that colonize roots and stimulate plant growth. Such bacteria and fungi have been applied to a wide range of agricultural species for the purpose of growth enhancement, including increased seed emergence, plant weight, crop yield and disease control (17, 21). The mechanisms of plant growth promotion by PGPR and PGPF have been reported, including plant hormone production (24, 25, 43), substrate degradation (mineralization) and suppression of deleterious microorganisms (18, 26).

In the past few years the role of volatile emissions from rhizobacteria in plant development has been widely studied. Ryu *et al.* (32) first reported a blend of airborne chemicals released from specific strains of PGPR, *Bacillius subtillis* GB03 and *Bacillius amyloliquefaciens* IN937a, which promoted the growth of *Arabidopsis thaliana* seedlings. Gutiérrez-Luna *et al.* (15) also reported that VOCs from some strains of *Bacillius* sp. have a growth promotion effect. While most studies have focused on the effect of VOCs released from PGPR and plant pathogens, little is known about the molecular mechanisms of response and resistance offered by PGPF-released VOCs.

Previously, different PGPF isolates such as *Phoma* sp. (GS8-3, GS8-1) and *Penicillium simplicissimum* (GP17-2) have been reported for their growth promotion effect (27, 28, 38, 39, 42); however, VOCs from these have not been analyzed. The first report regarding the growth promotion effect of VOCs produced by PGPF was by Yamagiwa *et al.* (44), who introduced a new PGPF, *Talaromyces wortmannii*, having a growth promotion effect on several plant species, such as *Brassica campestris*, *Arabidopsis thaliana*, *Phaseolus vulgaries*, *Nicotiana benthamiana* and *Cucumis sativas*. The major volatile component isolated from that PGPF was a terpenoid-like volatile, β-caryophyllene, which significantly promoted plant growth and induce resistance in turnip (44).

Considering that the fungi produce a wide range of VOCs (12) and VOCs produced from microorganisms play an important role in plant growth, we aimed to analyze the plant growth promotion effect of VOCs released from a previously reported PGPF, *Phoma* sp. GS8-3.

## Materials and Methods

### Fungal cultures

One hundred fungal isolates were used in this experiment. All of the isolates were obtained from the plant pathology laboratory of Gifu University. Airborne fungi were isolated from leaves of turf grass around Gifu city and soil-borne fungi were isolated from the rhizosphere of cucumber, tomato and leaf mustard. Most of the isolates were identified by sequence comparison in the ITS regions of the rRNA gene, including five of the selected fungi: *Cladosporium* sp. (D-c-4), *Ampelomyces* sp. (D-b-7, F-a-3), *Mortierella* sp. (U-c-1) and *Phoma* sp. (GS8-3) (data not shown). The fungal isolates were cultured on potato dextrose agar (PDA), and the periphery of actively growing cultures was cut with a cork borer of 5 mm diameter for use in the experiment. The fungal cultures were maintained on PDA slants and stored at 5°C.

### Preparation of Plant materials

Seeds of *Nicotiana tabacum* L. cv. Xanthi-nc were surface-sterilized (70% ethanol soaking for 2 min, followed by 5% sodium hypochlorite soaking for 2 min), rinsed (five times) in sterile distilled water, and placed on Petri dishes containing Murashige and Skoog salt (MS) medium (Wako) containing 0.8% agar, and the pH was adjusted to 5.7. The seeds were incubated in growth cabinets (LH-100S; Nihon ika kikai seisakusho) set to a 12-h light/12-h dark cycle at 25°C.

### Screening of fungal isolates showing plant growth promotion

Plastic Petri dishes (90×15 mm) containing a center partition (I plates; Atekuto) were prepared with 5 mL MS solid medium on one side and 5 mL PDA on the other side. Fourteen-day-old tobacco seedlings (10 seedlings per plate) were transferred to the MS solid medium side of the I plates. Treatments were performed by inoculating the I plates with a disk of fungal isolate in the center of PDA medium. Control was maintained by using PDA medium without a fungal disk. The plates were sealed with Parafilm and arranged in a randomized design within the growth cabinets and incubated at 25°C with a 12-h light/12-h dark photoperiod.

### Design of screening of fungal isolates

Test fungal isolates were selected randomly considering the origin of isolates and pattern of growth promotion. Among the seven test fungi, four were selected from the airborne fungal group: *Cladosporium* sp. (D-c-4), *Ampelomyces* sp (D-b-7 and F-a-3) and C-b-9 (unidentified), whereas the other three: *Phoma* sp. (GS8-3), E-a-2 (unidentified) and *Mortierella* sp. (U-c-1), were from the soil-borne fungal group. Considering the pattern of growth promotion effects, D-c-4 (*Cladosporium* sp.), GS8-3 (*Phoma* sp.), and D-b-7 (*Ampelomyces* sp.) were selected from the group of fungi that had a higher growth promotion effect, whereas F-a-3 (*Ampelomyces* sp.) was selected from the medium group, and unidentified E-a-2 and C-b-9 and U-c-1 (*Mortierella* sp.) were selected from the fungi having a lower growth promotion effect.

### Measurement of CO_2_ regulation by the test fungus

The test fungal isolates were inoculated into a 300 mL Erlenmeyer flask containing 100 mL PDA and cultured in an incubator set to 12-h light/12-h dark cycle for 7 d at 25°C. Three, 5, 7, 9, 12 and 14 d after inoculation, CO_2_ concentration in the jar was measured by a CO_2_ detector.

### Analysis of volatiles produced from a selected fungal isolate GS8-3 for plant growth promotion effect

The assay was performed in two Erlenmeyer flasks that were attached to a glass tube with adapters for air inlet and outlet. The first Erlenmeyer flask was prepared with 100 mL PDA medium and the second flask was prepared with 100 mL MS solid medium. The tobacco seedlings incubated for 14 d (20 seedlings per a flask) were transferred to the MS solid medium-containing flask. PGPF isolate GS8-3 was used as the test fungus and was incubated onto PDA medium in the Erlenmeyer flask. Air was passed one-way over the fungal culture to the plant culture at 10 mL min^−1^. In another set, a charcoal and silica-gel tube (SIBATA) was used as an absorbent of the volatile compounds produced by the test fungus to compare the effect of the compounds on plant growth. The absorbent was connected in the middle of the glass tube, which was attached to the fungal culture flask connected to the plant culture. Control was maintained by using PDA medium without fungal disk. The whole apparatus was incubated at 25°C with a 12-h light/12-h dark photoperiod for 14 d. The tube was replaced every third day.

### Measurement of atmospheric CO_2_ in vitro and analysis of its effect on plant growth

Three sets of I plates were used in this experiment. The I plates were prepared with 5 mL MS solid medium on one side and 5 mL PDA on the other side. In the first design, 14-day-old tobacco seedlings were transferred to the MS solid medium side (10 seedlings per plate) and the PDA side of the I plates was inoculated with a disk containing GS8-3. In the second design, the PDA side of I plates contained only PDA without fungus. In the third design, the PDA side of the I plates was inoculated with a disk containing GS8-3 but the MS solid medium did not contain plants. The I plates were then placed in an AnaeroPack rectangular jar (2.5 L) (Mitsubishi Gas Chemical, Tokyo, Japan) that contained an AnaeroPack MicroAero (Mitsubishi Gas Chemical). The AnaeroPack MicroAero is a non-disposable oxygen-absorbing and carbon dioxide-generating agent for use in an anaerobic jar. The experiment was performed under 7% (vol) preliminary CO_2_ concentration with the AnaeroPack MicroAero in the jar. The jar was placed in growth cabinets with a 12-h light/12-h dark cycle for 7 d at 25°C. Tobacco plants with and without fungus were also grown to compare plant growth in the jar without Anaeropack MicroAero. The CO_2_ concentration and plant growth were compared between the treatments with or without the Anaeropack MicroAero. There were five replicates for each treatment and the CO_2_ concentration in the jar was measured by a CO_2_ detector (New Cosmos Electric Co., Osaka, Japan) 1, 3, 5 and 7 d after treatment.

### Extraction and analysis of volatile metabolites

GS8-3 was cultured in 10 mL solid phase micro-extraction (SPME) vials (Supelco; Sigma-Aldrich Co., St Louis, MO, US) for 3, 5, 7 and 9 d. The volatile metabolites were extracted by headspace SPME during 30 min at 25°C. Polydimethylsiloxane/divinylbenzene (PDMS/DVB) (65 μm) fibers were used for volatile profiling. Fibers were obtained from Supelco and conditioned prior to analyses according to the manufacturer’s recommendations.

A Hewlett-Packard 5890 gas chromatograph (GC) equipped with a split injector HP-5 MS capillary column (30-m length, 0.25-mm inner diam.) was combined by direct coupling to a Hewlett-Packard 5972 A mass spectrometer (GC-MS). Working conditions were: injector 250°C, transfer line to MS system 250°C, oven temperature start 40°C, hold 2 min, increased from 40 to 200°C at a rate of 10°C min^−1^, and then from 200 to 250°C at a rate of 15°C min^−1^, hold 5 min; carrier gas (He) 1.0 mL min^−1^; the analytes were injected in split mode (1/10); electron impact ionization 70 eV. Peak areas of total ion current were used for comparison of volatile compound fractions. Compounds were identified using the US National Institute of Standards and Technology (NIST) Mass spectral Library or by comparison of retention times and spectra with those of authentic standards and Kovats retention indices with literature data.

### Analysis of plant growth-promoting effect of volatile organic compounds produced by PGPF isolate GS8-3

I plates were prepared with 5 mL MS solid medium on one side. Fourteen-day-old pre-germinated tobacco seedlings were transferred to the side of I plates. The compounds identified through GS-MS analysis were purchased (synthetic chemicals) to carry out the plant growth promotion test. The compounds were diluted in CH_2_Cl_2_ or the solvent alone was mixed with 0.1 lanolin, and 20 μL of the resulting suspension was applied to a sterile paper disk (1 cm diam.). Each of the compounds was tested for plant growth-promoting effect by placing 1.8×10^−4^ and 1.8×10^−2^ μg singly and in combination with the compounds, on sterile filter paper discs placed on the blank side of I plates. The plates were sealed with Parafilm and arranged in a randomized design within the growth cabinets and incubated at 25°C with a 12-h light/12-h dark photoperiod. There were four replications for each treatment and the experiments were repeated three times.

### Statistical Analysis

Data of growth promotion was analyzed by analysis of variance (ANOVA). The significance of the effect of fungal treatments was determined by the magnitude of the *F* value (*P*=0.05). When a significant *F* test was obtained for treatments, separation of the means was accomplished by Fisher’s protected least significant difference (LSD) test.

## Results

### Screening of fungal isolates showing plant growth promotion

**Fig. 1:**
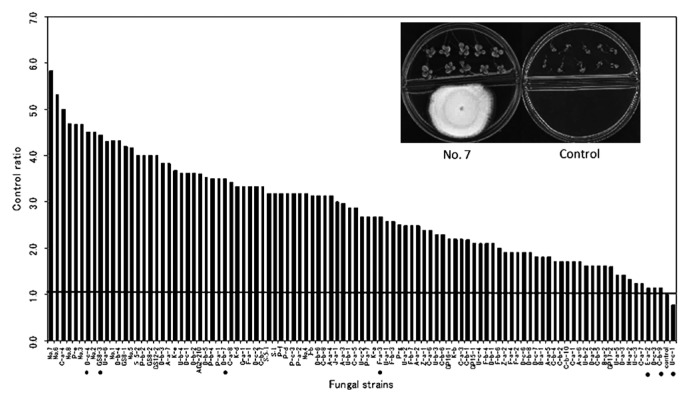
Analysis of growth promotion in tobacco with exposure to airborne chemicals released from 100 fungal isolates compared with control (PDA only). Representative example of 7-day-old tobacco seedlings grown on I plates with exposure to airborne fungal isolate (isolate No. 7) and PDA only are shown in inset. I-plates were prepared as a gnotobiotic system to avoid contamination. Figure shows the fresh weight of tobacco under different treatments with control ratio as fresh weight of the control set as one. Data are the mean of three independent experiments. Seven isolates (marked with black dots) were randomly selected as test fungi for the next experiment.

**Fig. 2:**
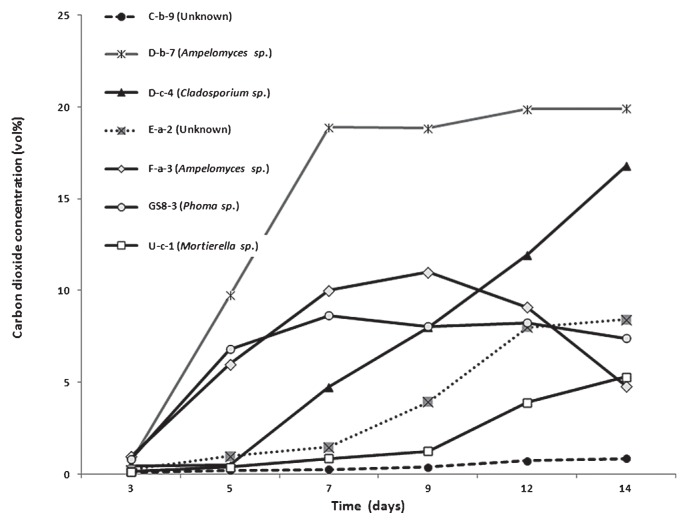
Growth of tobacco seedlings over 14 d with exposure to airborne chemicals released from selected fungal isolates compared with PDA alone (blank). There were four replicates for each treatment and the experiments were repeated three times. Data are the mean of three independent experiments. Different letters indicate significant differences between treatments according to Fisher’s LSD at *P*=0.05

One hundred fungal isolates were screened for their growth promotion effect on tobacco plants. Almost all of the fungal isolates were found to promote plant growth except for isolate U-c-1. Among them, 70 isolates were found to almost double plant growth compared to control treatment 7 d after transplanting (Fig. 1). Seven isolates, D-c-4 (*Cladosporium* sp.), D-b-7 (*Ampelomyces* sp.), F-a-3 (*Ampelomyces* sp.), GS8-3 (*Phoma* sp.), C-b-9 (unidentified air borne fungus), U-c-1 (*Mortierella* sp.) and E-a-2 (unidentified soil borne fungus), were randomly selected to rescreen their growth promotion effect in tobacco, maintaining the time course as 3, 5, 7, 10 and 14 d after treatment. All isolates showed significantly higher growth at 14 d compared to the control, triggering gradual growth promotion until 7 d and then a sharp increase of plant fresh weight (Fig. 2). U-c-1 was found to have a comparatively poor growth promotion effect while D-c-4 had the highest, validating the preliminary result in which this isolate belonged to the top group of isolates. In this experiment, I plates (Atekuto) were used and have a central partition that avoids physical contact between the fungus and the plant seedlings, allowing only airborne signal transmission.

### Measurement of CO_2_ production by the test fungus and analysis of its effect on plant growth

**Fig. 3:**
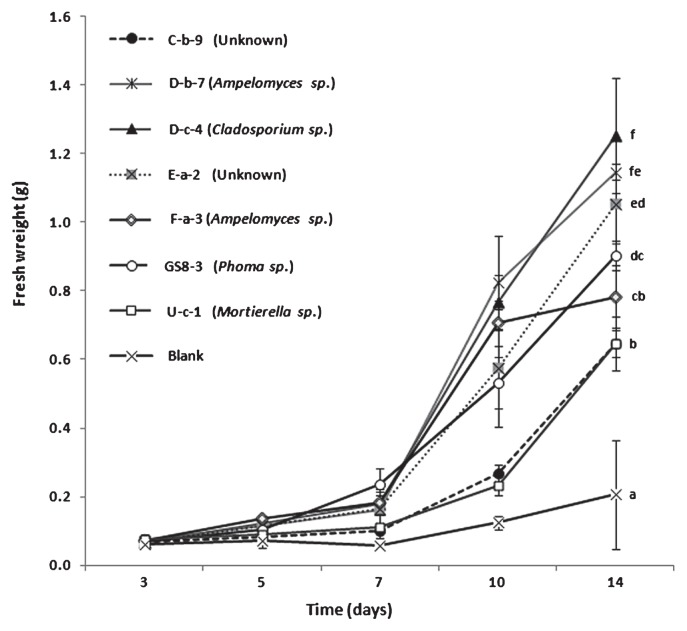
Production of CO_2_ by selected fungal isolates during the 14-d growth period. The test fungal isolates were inoculated into a 300 mL Erlenmeyer flask containing 100 mL PDA and cultured in an incubator set to 12-h light/12-h dark cycle for 7 d at 25°C. Three, 5, 7, 9, 12 and 14 d after inoculation, CO_2_ concentration was measured by a CO_2_ detector. Data are the mean of three independent experiments.

Since CO_2_ plays an important role in plant growth it is necessary to measure CO_2_ regulation by the test fungal isolates and their role in plant growth. Test isolates showed a variable trend in CO_2_ production. D-b-7 and D-c-4 showed the highest production of CO_2_ 14 d after inoculation, indicating a positive correlation between the increase of CO_2_ regulation and growth promotion of tobacco, although the patterns were different (Fig. 3 and 2); however, F-a-3 showed a higher rate of CO_2_ production for the first 9 d but subsequently it gradually decreased. In the case of GS8-3, CO_2_ concentration showed an increase for the first 7 d but after that it marginally decreased. In the case of U-c-1 and E-a-2, a slowly increasing CO_2_ concentration pattern was noticed whereas C-b-9 was notable in showing an exceptionally slow increase in CO_2_ production. These results suggest that F-a-3, C-b-9 and GS8-3 could promote the growth of tobacco 14 d after inoculation despite the decrease in CO_2_ production. Among the seven fungi, GS8-3 was selected for further analysis because *Phoma* sp. GS8-3 has previously been reported as a PGPF, as well as a biocontrol agent (27, 28, 38, 39, 42).

### Analysis of volatile substances produced from selected fungal isolate for plant growth promotion effect

**Fig. 4:**
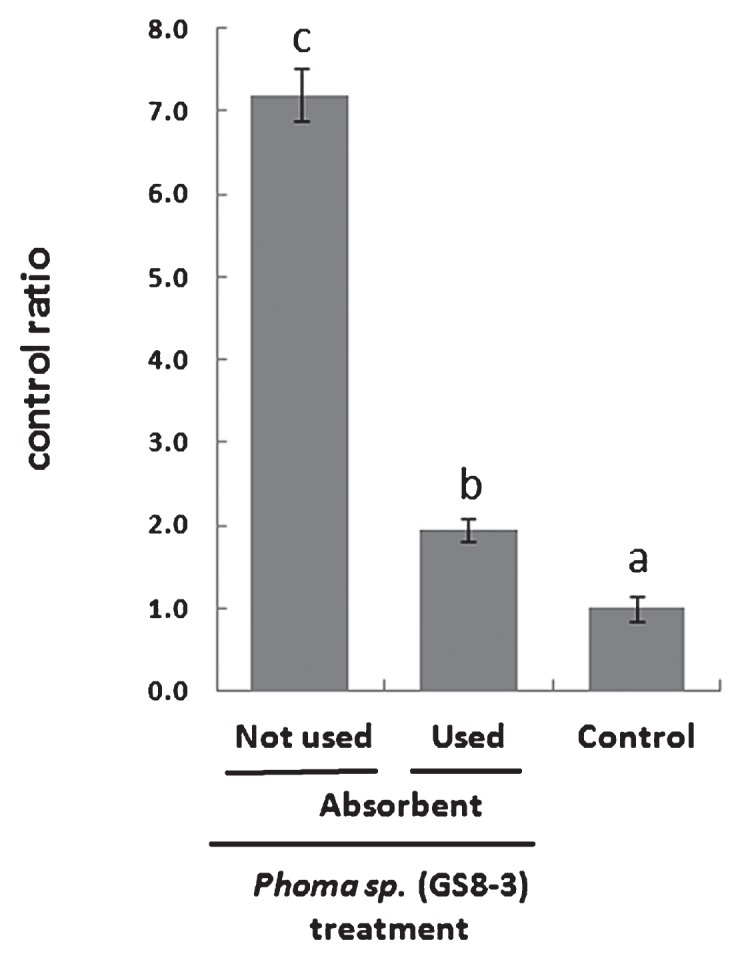
Growth promotion effect of volatile substances of *Phoma sp.* (GS8-3) in tobacco. PGPF *Phoma sp.* (GS8-3) was used as test fungus. Charcoal and silica-gel tube, which absorbs volatiles as soon as they are produced, was used to block the flow of volatile compounds toward the plant flasks and used for comparison. Control treatment was maintained using PDA only inside the flask without any fungal isolate. Data show fresh weight of tobacco under different treatments with the control ratio as fresh weight of the control set as one. Values are the means of three independent trials. Different letters on the bars indicate significant differences between treatments according to Fisher’s LSD at *P*=0.05.

To confirm the growth promotion effect of the volatile chemicals released from the test fungal isolate GS8-3, another experiment was performed using an absorbent of volatile substances. GS8-3-inoculated seedlings in which an absorbent was not used showed more than 7 times growth promotion whereas fungus-inoculated plants where an absorbent was used showed 1.5 times growth promotion over the control (Fig. 4). This result confirms the positive effect of airborne chemical signaling produced by GS8-3 on plant growth.

### Measurement of atmospheric CO_2_ in vitro and analysis of its effect on plant growth

**Fig. 5:**
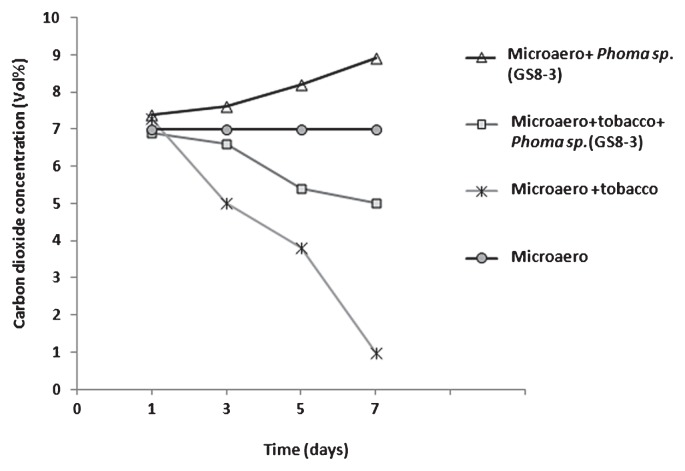
Concentrations of CO_2_ *in vitro* under MicroAero conditions with or without tobacco plants and/or *Phoma sp.* (GS8-3). Three sets of I plates were used in this experiment. In the first set, 14-day-old tobacco seedlings were transferred to MS media with PDA media on the other side inoculated with *Phoma sp.* (GS8-3). Second and third sets were prepared with fungus or plants only. The I plates were placed in an AnaeroPack rectangular jar with 7% (vol) preliminary CO_2_ concentration by AnaeroPack MicroAero. The jar was placed in a growth cabinet set to a 12-h light/12-h dark cycle for 7 d at 25°C. There were five replicates for each treatment and CO_2_ concentration in the jar was measured by CO_2_ detector 1, 3, 5 and 7 d after treatment.

Atmospheric CO_2_ was measured *in vitro* using the AnaeroPack MicroAero to identify the relation of plant growth with the CO_2_ level *in vitro*. The experiment was performed with 7% (vol) CO_2_ concentration preliminarily kept in a jar with an AnaeroPack MicroAero. In the case of GS8-3 only, the CO_2_ concentration gradually increased and reached 7% (vol) to 9% (vol) in the jar after 7 d of cultivation, whereas with a tobacco plant only, the CO_2_ concentration rapidly decreased after 3 d and was detected at 1% (vol) in the jar 7 d after planting (Fig. 5). When tobacco plants were cultivated in the same jar with GS8-3 under MicroAero, the CO_2_ concentration gradually decreased to 5% (vol) after 7 d of cultivation.

**Fig. 6:**
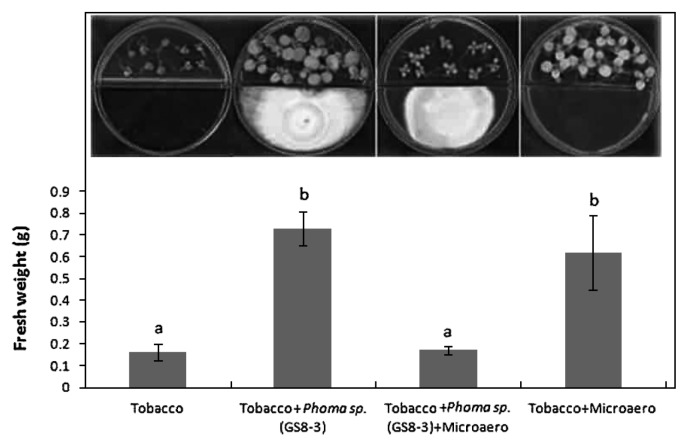
Growth promotion of tobacco seedlings under MicroAero conditions and/or *Phoma sp.* (GS8-3). Tobacco seedlings were grown for 14 d after treatment: from left, the seedlings grew alone (blank), with *Phoma sp.* (GS8-3), with *Phoma sp.* (GS8-3) under MicroAero conditions, or under MicroAero conditions without *Phoma sp.* (GS8-3). There were four replicates for each treatment and the experiments were repeated three times. The data are the means of three independent experiments. Bars marked with the same letters are not significantly different according to Fisher’s LSD at *P*=0.05.

The growth of tobacco seedlings was compared between different jars with or without the fungus and MicroAero conditions (Fig. 6). Fresh weight (g) of tobacco plants was significantly increased when cultivated under MicroAero conditions compared with the jar without MicroAero. The highest plant growth was found in tobacco plants treated with the fungus only, which is similar to the plants cultivated with MicroAero only. In contrast, plant growth was found to be very poor and the leaves had become slightly bleached when tobacco plants were treated with GS8-3 and cultivated under MicroAero conditions. Furthermore, the growth of GS8-3 in the jar with tobacco plants under MicroAero conditions seemed poor compared to that in the jar without an AnaeroPack MicroAero (Fig. 6).

### Extraction and analysis of volatile metabolites regulated from test fungus

Fifteen volatile organic compounds were extracted from PGPF GS8-3 using SPME-coated PDMS/DVB fibers. Among these, 14 were identified as C4-C8 hydrocarbons including alcohols (2-methyl-propanol, 3-methyl-butanol, 1-hexanol, 2-heptanol, 4-methyl-phenol, phenyl ethyl alcohol), carboxylic acids (acetic acid, methacrylic acid and tiglic acid), ketones (2-hexanone, 2-heptanone, 3-hydroxy-2-butanone/acetoin) and their ester (isobutyl acetate) (Table 1). To investigate the relationship between mold growth and the release of fungal volatile substances with time, the volatiles were extracted from different sets of fibers at 3, 5, 7 and 9 d of growth. GS8-3 produced 2-methyl-propanol and 3-methyl-butanol as the main volatile organic components during the culture periods; however, the number and concentration of the volatiles produced by GS8-3 differed with increasing age of the fungus.

### Effect of synthetic VOCs on plant growth

Synthetic VOCs that were identified from GS8-3 in 3- and 5-d cultures were tested for their growth promotion effects at four concentrations individually and using mixtures of two of them. In addition, other two VOCs (2,3-butanediol and 1-octen-3-o1), which were previously identified to have a growth promotion effect on *Arabidopsis* by other researchers were also chosen to compare their effects on tobacco. Mixture-1, which included VOCs, 2-methyl-propanol:3-methyl-butanol:methacrylic acid:isobutyl acetate (30:60:7:3), extracted from GS8-3 at 3 d showed a 1.4 times significant increase in the fresh weight of tobacco over solvent control at 1.8×10^−2^ μg concentration (Table 2). In addition, mixture-2 (at 1.8×10^−2^ μg), which included acetic acid:2-methyl-porpanol:acetoin:3-methyl-butanol:methacrylic acid:isobutyl acetate:tiglic acid:phenylethyl alcohol (14:20:6:46:9:2:2:3) and methacrylic acid (at 1.8×10^−4^ μg), acetic acid (at 1.8×10^−4^ μg) and tiglic acid (at 1.8×10^−4^ μg) individually showed noticeable favorable effects on growth promotion, although they were not significant. Fresh weight of tobacco varied at different concentrations of synthetic VOCs. At high concentrations, such as at 1.8 and 1.8×10^2^ μg, most of the compounds caused bleaching of the cotyledon leaves (data not shown).

## Discussion

We investigated 100 airborne and the soil-borne fungal isolates for their growth promotion effects on tobacco plants, and 70 isolates were found to almost double plant growth compared to the control treatment (Fig. 1). Among these, seven randomly selected isolates were rescreened, maintaining the time course, and were found to have significantly higher growth promotion effects. In this study, we maintained air-tight cultivation using I plates, which restrict physical contact between the fungus and the plant seedlings and allow only gaseous exchange. This result suggests that the volatile or gaseous compounds released from the fungal strains have growth promotion effects on tobacco plants and our results support the data of Ryu *et al.* (32). These fungi included *Phoma* sp. GS8-3, which has previously been reported as a PGPF, as well as a biocontrol agent (27, 28, 38, 39, 42), and was used as a test fungus in the following experiments. In another test, plant growth was found to more than double in the case of GS8-3-treated seedlings without using absorbents compared to control treatment or GS8-3-treated plants where charcoal and silica-gel tube absorbents were used (Fig. 4). These materials adsorbed the volatiles as soon they were produced by the organism and blocked the transfer of volatiles to the seedlings. Our method supports the method of Fernando *et al.* (14). In this experiment, air-tight cultivation plates were used, suggesting that the gaseous atmosphere was normalized or CO_2_ concentration was elevated through the fungal colony or culture. Thus, CO_2_ produced by the fungus inside the chamber might affect plant growth. Many reviews have also been published on the increased growth of plant species by improved CO_2_ supply (5, 7, 9, 31, 40). Consequently, in this study we also considered the effect of the amount of CO_2_ *in vitro* during the analysis of the growth promotion effects of PGPF-released volatile metabolites in tobacco. Although previous researchers (32, 44) who worked on the plant growth promotion effect of VOCs from microorganisms did not mention the involvement of CO_2_, we located a report (12) where considerable amounts of CO_2_ were recovered along with the VOCs during the profiling of some PGPR. Thus, we checked CO_2_ production by the previously mentioned seven test fungi until 14 d of culture. Data showed that among the isolates, D-b-7 and D-c-4 gradually increased CO_2_ production, indicating a positive correlation between the increase of CO_2_ regulation and growth promotion of tobacco (Fig. 2 and 3). Thus, we assumed that CO_2_ might play an important role in the plant growth promotion effect in D-b-7 and D-c-4; however, in other isolates, including GS8-3, no such correlation was found as GS8-3 could still increase plant growth significantly in spite of the decrease in CO_2_ production after 7 d. Thus, we could distinguish the effect of VOCs in *Phoma* sp. GS8-3 rather than the effect of CO_2_. Moreover, among the isolates, only *Phoma* spp. have been reported as effective PGPF for many crop species from a detailed study in our laboratory over several years (18). Therefore, in this work we chose *Phoma* sp. GS8-3 as the test fungus to analyze the effects of VOCs released from this fungus for better understanding its growth promotion mechanisms. In a further experiment, we measured the atmospheric CO_2_ concentration in the presence or absence of GS8-3 and its effect on plant growth *in vitro*. We used AnaeroPack MicroAero for CO_2_ supplement *in vitro*, *i.e.*, a non-disposable oxygen-absorbing and carbon dioxide-generating agent for use in an anaerobic jar. The results showed that level of CO_2_ inside the jar was increased when inoculated with GS8-3 with or without plants until 7 d after inoculation (Fig. 5). Another set of experiments was performed without using MicroAero and the fresh weight of tobacco plants was measured and compared under both conditions 14 d after planting. Fresh weight (g) of tobacco plants was significantly increased when cultivated under MicroAero conditions compared with the jar without MicroAero (Fig. 6). This result supports the findings of Haisel *et al.* (16) as they reported that tobacco plantlets better supplied with CO_2_ had a high net photosynthetic rate, and a low transpiration rate and stomatal conductance; however, the highest plant growth was found in tobacco plants treated with GS8-3 alone in the absence of MicroAero, although it was statistically similar to the plants cultivated with MicroAero only. From the previous experiment (Fig. 3) we found that GS8-3 decreases CO_2_ production 7 d after inoculation. This result indicates that aside from CO_2_, GS8-3 produces VOCs that could promote plant growth. Plant growth was notably poor and leaves became minimally bleached when tobacco plants were treated with GS8-3 and cultivated under MicroAero conditions (Fig. 6). In addition, the growth of GS8-3 in the jar seemed poor compared to that in the jar without an AnaeroPack MicroAero (Fig. 6 Inset). It may be the cause that excess CO_2_ inhibited the growth of the fungi and changed the gaseous content inside the chamber by reacting with the VOCs. Previous reports (6, 41) indicated that higher concentrations (more than 5% increases in concentration) of CO_2_ inhibit the growth of microorganisms, especially soil-borne fungi. The altered gaseous atmosphere might be the cause of the growth retardation and bleaching symptoms of tobacco seedlings; however, the growth promotion effect on tobacco by GS8-3 alone was higher than that by CO_2_ supply using MicroAero.

In the next step, we separated the volatile components emitted from GS8-3 at different culture times by gas chromatography and identified them by mass spectrometry. Identified VOCs belonged mostly to four classes of C4–C8 hydrocarbons, where 2-methyl-propanol and 3-methyl-butanol were mostly found in considerable concentrations at all fungal ages (Table 1). Compounds of these characteristic metabolites were detected as indicator substances for mold growth (4). These two components were previously extracted from PGPR (12). Volatiles varied in number and amount by the age of the fungus. Among the identified VOCs, acetoin (3-hydroxy-2-butanone) has been discussed in many reports (12, 32, 33) for its growth-promoting and ISR-triggering ability in *Arabidopsis* when released from PGPR. We opted to analyze all the VOCs extracted at 3 and 5 d of GS8-3 culture for the growth promotion effect, as the rest of the compounds have been found in trace amounts. Aside from these, 2,3-butanediol (32), and 1-octen-3-o1 (20, 30, 36) have also been checked in tobacco as these two metabolites were previously reported to promote growth and to induce a defense response in *Arabidopsis*. Synthetic VOCs and their mixtures were analyzed at four concentrations. Mixture -1 (2-methyl-propanol:3-methyl-butanol:methacrylic acid:isobutyl acetate in 30:60:7:3 ratio, respectively) showed the greatest level of growth promotion (1.4 times) compared to the control (Table 2).Mixture-2(aceticacid:2-methyl-porpanol:acetoin:3-methyl-butanol:methacrylic acid:isobutyl acetate:tiglic acid:phenylethyl alcohol in 14:20:6:46:9:2:2:3 ratio, respectively) also showed a better result than control. This supports the findings of Ryu *et al.* (32) that a better growth promotion effect is seen from all VOC blends. Although the VOCs did not show significant individual growth promotion effects, a few, such as methacrylic acid, acetic acid and tiglic acid, still showed a good control ratio. Yamagiwa *et al.* (44) also reported a similar growth promotion effect of volatile β-caryophyllene in turnip; however, we failed to notice a positive effect of 2, 3-butanediol and 1-octen-3-o1 on tobacco. The growth-stimulating ability of VOCs probably differ according to plant species. As the fresh weight of tobacco plants varied at different concentrations of synthetic VOCs, from our observations, VOCs at lower concentrations showed better growth promotion than at higher concentrations.

Previously, we mentioned that plant growth-promoting microorganisms promote plant growth by producing growth-regulating hormones (24, 25, 43), mineralizing nutrient substrates (18) and suppressing deleterious microorganisms (12, 14, 20, 33). Ryu *et al.* (32) revealed the possible involvement of PGPR-regulated VOCs in the growth regulatory signaling pathways by using different mutant plants. They also speculated the possibility of using PGPR VOCs in other cultivation methods other than air-tight cultivation. We also analyzed the growth promotion effects of PGPF-produced VOCs in an open air-cultivation system, but in our case, VOCs were not found to be effective growth inducers in the open-air system (data not shown). In this report, we have tried to elucidate the potential role of PGPF-regulated VOCs in the range of growth regulatory mechanisms. We found that *Phoma* sp. GS8-3 could induce growth promotion in tobacco in an airtight cultivation system, suggesting its simultaneous participation in the growth promotion effect of plant growth-promoting fungi; however, the involvement of PGPF-released VOCs in the growth regulatory signaling pathways remains to be determined.

## Figures and Tables

**Table 1:** VOCs extracted from the PGPF isolate *Phoma sp.* (GS8-3) after 3, 5, 7 and 9 d

Compounds	RI	Peak area (%)			

		3 d	5 d	7 d	9 d
Acetic acid		0	13.7	0	0
2-Methyl-propanol	621	28.9	19.8	9.4	17.5
3-Hydroxy-2-butanone/Acetoin	710	0	6.0	0	0
Unknown	713	0	0	0	3.2
3-Methyl-butanol	740	62.1	45.9	83.5	59.6
Methacrylic acid	761	7.0	8.8	0	7.1
Isobutyl acetate	789	2.0	1.5	0	0
2-Hexanone	811	0	0	0	2.1
Octane	801	0	0	0	1.9
Tiglic acid	849	0	1.6	0.4	1.0
1-Hexanol	870	0	0	0	3.6
2-Heptanone	894	0	0	0.4	2.3
2-Heptanol	902	0	0	0.4	0
4-Methyl-phenol	1080	0	0	3.2	0
Phenyl ethyl alcohol	1126	0	2.7	2.7	0
Total		100	100	100	100

RI = Retention index. Compounds identified based on the comparison of retention index and mass spectra with NIST database.

**Table 2:** Plant growth promotion effect on tobacco with exposure to volatile organic compounds (VOCs) released from PGPF isolate *Phoma sp.* (GS8-3).

VOCs	Concentration (μg)	
	1.8×10^−4^	1.8×10^−2^
2-Methyl-propanol	1.0	0.9
3-Methyl-butanol	0.9	1.1
Phenyl ethyl alcohol	1.1	1.0
3-Hydroxy-2-butanone	1.0	0.8
2,3-Butanediol^d^	0.9	0.9
1-Octen-3-ol^e^	1.0	1.0
Methacrylic acid	1.2	1.1
Isobutyl acetate	1.0	1.0
Acetic acid	1.2	1.0
Tiglic acid	1.3	1.0
Mixture 1^b^	1.0	1.4^a^
Mixture 2^c^	0.9	1.2

Tobacco seedlings were treated for 14 d with VOCs. Table shows fresh weight of treated plants with control ratio as the fresh weight of control set as 1.

a indicates significant difference at *P* <0.05 (LSD).

b mixture that duplicated volatiles produced by GS8-3 at 3 d; 2-methyl-propanol:3-methyl-butanol:methacrylic acid:isobutyl acetate=30:60:7:3.

c Mixture -2 duplicated volatiles produced by GS8-3 at 5 d; acetic acid:2-methyl-propanol:3-hydroxy-2-butanone:3-methyl-butanol:methacrylic acid:isobutyl acetate:tiglic acid:phenyl ethyl alcohol=14:20:6:46:9:2:2:3.

d,e VOCs previously reported by other researchers were used to compare growth promotion effects.
